# CRISPR/Cas9 editing of the MYO7A gene in rhesus macaque embryos to generate a primate model of Usher syndrome type 1B

**DOI:** 10.1038/s41598-022-13689-x

**Published:** 2022-06-16

**Authors:** Junghyun Ryu, John P. Statz, William Chan, Fernanda C. Burch, John V. Brigande, Beth Kempton, Edward V. Porsov, Lauren Renner, Trevor McGill, Benjamin J. Burwitz, Carol B. Hanna, Martha Neuringer, Jon D. Hennebold

**Affiliations:** 1 Division of Reproductive and Developmental Sciences, Oregon National Primate Research Center, Oregon Health and Science University, Beaverton, OR 97006 USA; 2 Assisted Reproductive Technologies Core, Oregon National Primate Research Center, Oregon Health and Science University, Beaverton, OR 97006 USA; 3 Department of Otolaryngology, Oregon Hearing Research Center, Oregon Health and Science University, Portland, OR 97239 USA; 4 Division of Neuroscience, Oregon National Primate Research Center, Oregon Health and Science University, Beaverton, OR 97006 USA; 5 Department of Ophthalmology, Casey Eye Institute, Oregon Health and Science University, Beaverton, OR 97006 USA; 6 Vaccine and Gene Therapy Institute, Oregon Health and Science University, Beaverton, OR 97006 USA; 7 Department of Obstetrics and Gynecology, Oregon Health and Science University, Portland, OR 97239 USA; 8 Present Address: Division of Biological Sciences, University of Montana, Missoula, MT 59812 USA; 9 Present Address: University of Texas Southwestern Medical School, 5323 Harry Hines Blvd, Dallas, TX 75390 USA

**Keywords:** Developmental biology, Genetics, Molecular biology, Neuroscience, Diseases

## Abstract

Mutations in the *MYO7A* gene lead to Usher syndrome type 1B (USH1B), a disease characterized by congenital deafness, vision loss, and balance impairment. To create a nonhuman primate (NHP) USH1B model, CRISPR/Cas9 was used to disrupt *MYO7A* in rhesus macaque zygotes. The targeting efficiency of Cas9 mRNA and hybridized crRNA-tracrRNA (hyb-gRNA) was compared to Cas9 nuclease (Nuc) protein and synthetic single guide (sg)RNAs. Nuc/sgRNA injection led to higher editing efficiencies relative to mRNA/hyb-gRNAs. Mutations were assessed by preimplantation genetic testing (PGT) and those with the desired mutations were transferred into surrogates. A pregnancy was established from an embryo where 92.1% of the PGT sequencing reads possessed a single G insertion that leads to a premature stop codon. Analysis of single peripheral blood leukocytes from the infant revealed that half the cells possessed the homozygous single base insertion and the remaining cells had the wild-type *MYO7A* sequence. The infant showed sensitive auditory thresholds beginning at 3 months. Although further optimization is needed, our studies demonstrate that it is feasible to use CRISPR technologies for creating NHP models of human diseases.

## Introduction

USH is the leading cause of congenital deaf-blindness in humans with a prevalence of 4 to 17 per 100,000^1,2^. This syndromic form of retinitis pigmentosa (RP) is characterized by deficits in hearing, balance and vision^3–5^. Depending on the disease phenotype and severity of symptoms, USH can be classified into three subtypes. USH1 patients show congenital deafness, vestibular dysfunction, and the onset of retinal degeneration within 10 years from birth. USH2 patients show moderate to severe congenital deafness and their onset of RP is delayed until late adolescence. USH3 patients show gradual hearing loss and variable onset of RP^4,5^. Mutations in the *MYO7A* gene are a cause of USH1B. The protein encoded by the *MYO7A* gene, myosin VIIA, is an unconventional myosin motor protein expressed in several tissues, including the inner ear and eye^5^.

Gene augmentation therapy and cell transplantation are potential treatments for individuals with USH, including USH1B. Gene augmentation therapy is challenging due to the large size of the *MYO7A* gene, requiring the development of dual-AAV vectors and other strategies for gene delivery^6–8^. While initial results with dual-AAV vectors are promising, questions still persist about the design of optimal gene therapies for replacement of large genes such as *MYO7A*, timing of treatment, proper immunosuppression, and the optimal surgical method for delivery of gene therapy vectors or transplanted cells in humans^9,10^. Within the inner ear, several gene therapy strategies were successfully utilized in mouse models with cochlear hair cell defects; however, it is predicted that these interventions would need to be delivered prenatally in humans^11^. Accordingly, an animal model with greater translatability to humans is necessary to further pursue the full range of potential therapeutic technologies.

Rhesus macaques share a high degree of genetic, physiological, anatomical, and behavioral similarities with humans. As such, they represent an ideal human surrogate for testing and optimizing new therapies. NHP models of Usher syndrome are particularly urgently needed because rodent models of USH1 fail to show a retinal degeneration phenotype due to differences in the cellular distribution of *MYO7A* expression. In humans and NHPs, *MYO7A* is expressed in pigment epithelium cells and photoreceptors. *MYO7A* expression in the mouse only occurs in pigment epithelium, which likely explains the lack of significant vision loss in *Myo7a* null mutant mice^12,13^. However, the production of transgenic NHPs to serve as human disease models has largely been unfeasible due to inefficiencies in technologies such as somatic cell nuclear transfer or chimeric NHP production using stem cells^14–17^. Through the use of the programmable nuclease CRISPR/Cas9, these issues can be circumvented by direct injection of Cas9 and the relevant gRNA into zygotes. While injection of CRISPR/Cas9 in NHP embryos has shown promise in creating disease models, using such an approach still faces significant hurdles^18^. These include optimizing injection reagents and injection timing for maximum gene targeting efficiency while prescreening transferable embryos for the desired edit. A previous study demonstrated that simultaneous delivery of three gRNAs targeting regions in close proximity to one another, as compared to a single gRNA, increased the creation of insertion or deletion (indel) mutations leading to a higher knockout (KO) efficiency in cynomolgus macaque embryos^19^. Moreover, different formulations of gRNAs and Cas9 (i.e., mRNA versus protein) are available that may impact embryo editing efficiencies. A commonly used gRNA system includes a Hyb-gRNA formulation that requires annealing of crRNA with a tracrRNA to form a functional gRNA. In contrast, synthetic single gRNAs possess both Cas9 binding and target gene protospacer sequences, typically incorporating endonuclease resistant nucleotides that prevent its degradation. Currently, limited information exists regarding the efficacy of these different formulations of gRNA/Cas9 for editing rhesus macaque embryos.

To generate an USH1B NHP model, exon 3 of the rhesus macaque *MYO7A* gene was targeted to introduce indels. Exon 3 codes for the region of the protein that is upstream of the motor domain of myosin VIIA, which is a major site of pathogenic mutations in humans^20,21^. After confirmation of gRNA efficiency in vitro in the rhesus macaque CMMT cell line, CRISPR/Cas9 reagents were injected into rhesus macaque zygotes. Our results demonstrate that Nuc/sgRNA injection possessed significantly greater *MYO7A* editing rates as determined in trophectoderm (TE) biopsies and arrested embryos relative to the injection of mRNA/hyb-gRNA. However, the use of Nuc/sgRNA resulted in a significantly lower blastocyst development rate relative to the mRNA/hyb-gRNA injections. Six embryos were transferred to five surrogates, resulting in the birth of one *MYO7A* KO female macaque named “Mya”. Single-cell genotyping results showed that Mya carries a mosaic mutation within exon 3 of the *MYO7A* gene. Auditory thresholds at 3–12 months were consistent with age-matched controls, and retinal structure and function also appeared normal at all ages tested. These results demonstrate the feasibility of a gene-editing approach and assisted reproductive technologies for the creation of valuable NHP disease models, although further studies are needed to improve overall efficiencies, reduce the incidence of mosaicism, and produce live births of fully edited animals.

## Results

### In vitro validation of *MYO7A* gRNAs

**Figure 1:**
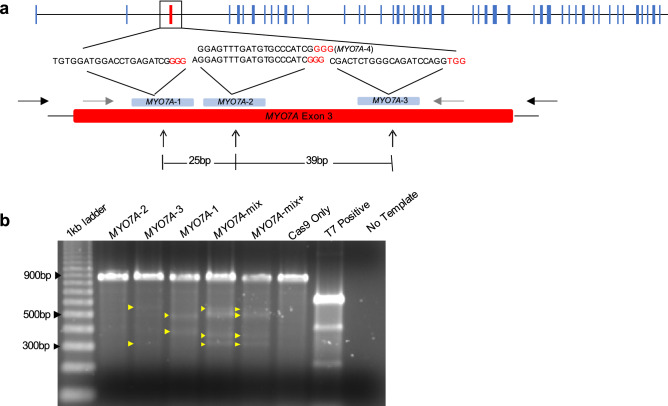
*MYO7A* exon 3 targeting strategy and assessment of gRNA targeting efficiency. (**a**) Using gRNA design programs described in the “Methods” section, four gRNAs were identified that correspond to exon 3 of the rhesus macaque *MYO7A* gene. Two gRNAs (#2 and #4) had overlapping targeting sites and gRNA #4 was not used further. Black letters represent the gRNA protospacer/target sequence and red letters indicate the PAM sequence. Gray arrows represent the primers used to amplify the flanking region of the target site. Black arrows were primers used to generate the PCR amplicons needed to perform the T7E1 editing assay. The distances between sgRNA indicated PAM to PAM distances. (**b**) Targeting efficiency was assessed using the rhesus macaque CMMT cell line, where each gRNA was tested separately and as a mixture (MYO7A-mix). Yellow arrowheads indicate the digested PCR amplicons by CRISPR/Cas9. MYO7A-mix + contained 50 ng of wild-type amplicon mixed with sample amplicon before performing the T7E1 assay. The negative control (–) contains Cas9 protein without gRNA, and the positive control is provided as part of the Integrated DNA Technologies Surveyor kit used to perform the T7E1 assay. PCR product size was 888 bp, and expected cleavage products were 500 bp and 388 bp for MYO7A-1, 524 bp and 364 bp for MYO7A-2, and 564 bp and 324 bp for MYO7A-3.

Four gRNAs targeting exon 3 of *MYO7A* were developed and initially tested for their targeting efficiency in vitro. Cas9 protein and each hyb-gRNA were combined with PCR amplicons containing the wild-type *MYO7A* exon 3 target site. All four gRNAs successfully generated double-stranded DNA breaks (DSB) on wild-type *MYO7A* amplicons (Supplementary Fig. 1). Three out of four hyb-gRNAs (#1 through #3) were subsequently tested in rhesus macaque mammary gland CMMT cells and #4 was excluded because the target sequences of gRNAs #2 and #4 overlapped by 19 bp (Fig. 1a). In CMMT cells, hyb-gRNA #1 and #3 resulted in the generation of indels in the *MYO7A* gene based on a T7E1 assay. Limited editing was observed with hyb-gRNA #2. However, when hyb-gRNA #1, #2, and #3 were co-transfected with a Cas9 expressing plasmid into CMMT cells, robust indel formation in *MYO7A* exon 3 was observed (Fig. 1b).

### Assessing targeting efficiency of different Cas9 and gRNA platforms in rhesus macaque embryos

Based on the validation of editing in CMMT cells, hyb-gRNAs comprised of a mixture of gRNAs 1 through 3 and Cas9 mRNA were injected into rhesus macaque zygotes (N = 53) to introduce indels in the *MYO7A* gene (Table 1). Out of 11 (20.75%) blastocysts and 33 (62.26%) arrested embryos, 36 samples (8 biopsies, 28 arrested embryos) successfully underwent whole genome amplification (WGA) and were genotyped by next-generation sequencing (NGS). NGS analysis showed 38.8% of embryos contained at least one edited allele. Nuc/sgRNAs for target sites #1, #2, and #3 were subsequently injected in a total of 234 embryos, of which 200 (85.47%) were arrested prior to forming a blastocyst and 26 (11.11%) became blastocysts. A total of 211 samples (19 biopsies, 192 arrested cleavage stage embryos) underwent successful WGA and were genotyped. Blastocyst biopsies and arrested embryos obtained after Nuc/sgRNA protein injection had an indel rate that was higher than that of mRNA/hyb-gRNA injected embryos (76.3% versus 38.8%, respectively; p < 0.01). Of the embryos observed to possess *MYO7A* indels after Nuc/sgRNA injection, a higher percentage (65.21%) were fully edited (i.e., no wild-type sequence detected) relative to edited embryos obtained following hyb-gRNA/Cas9 mRNA injection (28.5%; p < 0.01) (Table 1). Injection of Nuc/sgRNA resulted in a higher embryo arrest rate than mRNA/hyb-gRNAs injection (p < 0.0011; Table 1). Although the injection of mRNA/hyb-gRNA resulted in a blastocyst formation rate (20.75%) that was higher than that observed using Nuc/sgRNA (11.11%), the difference was not statistically significant (p = 0.0697).

**Table 1:** Comparison of the targeting efficiency using hyb-gRNA (mRNA/hyb-gRNA) and Cas9 mRNA with Cas9 protein and sgRNA (Nuc/sgRNA).

Injection material	# Total oocytes	# Fertilized oocytes (%^a^)	# Arrested embryos (%^b^)	Blastocysts (%^c^)	# Embryos analyzed^d^	# Embryos edited (%^e^)	# Fully edited embryos^f^ (%^g^)
mRNA/hyb-gRNA	125	53 (42.4%)	33 (62.26%)	11 (20.75%)	36	14 (38.88%)	4 (28.5%)
Nuc/sgRNA	409	234 (57.21%)	200 (85.47%)*	26 (11.11%)	211*	161 (76.3%)*	105 (65.21%)*

*p < 0.01, Nuc/sgRNA versus Cas9 mRNA/hyb-gRNA.

^a^% = number of cleavage stage embryos/total number of oocytes that underwent IVF X 100.

^b^% = number of arrested embryos/number of fertilized oocytes X100.

^c^% = number of blastocysts/number of fertilized oocytes X 100.

^d^Includes both arrested embryos and TE cell biopsy samples from blastocysts.

^e^% = number of any edited embryo biopsies and arrested embryos/total number analyzed X 100.

^f^Includes those arrested embryos and TE biopsies that possess 100% mutated sequence reads.

^g^% = number of 100% mutated sample/embryo edited X 100.

### Transfer of edited rhesus macaque embryos

**Figure 2:**
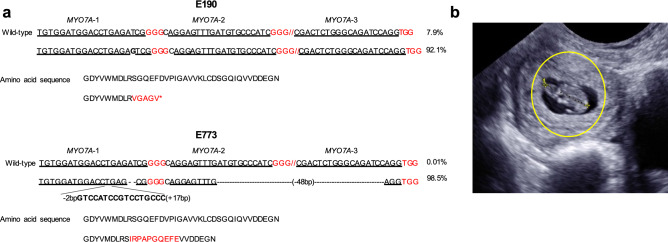
*MYO7A* genotype of TE biopsies from two embryos transferred into a recipient animal and resulted in a singleton pregnancy. (**a**) TE cell genotyping results of two expanded blastocysts selected for transfer to surrogate females. Double slashes denote omitted DNA sequence. Genotyping by NGS revealed E190 possessed a single G insertion in exon 3 of the *MYO7A* gene in 92.1% of sequencing reads, whereas 98.5% of the sequencing reads from E773 possessed an insertion and deletion in the target region. The single G insertion observed in E190 generates a premature stop codon (*). The mutation observed in E773 included a 50 bp deletion and a 17 bp insertion, with the net result being a change of 10 amino acids. (**b**) Ultrasound confirmed a singleton pregnancy at 28 days after transfer of E190 and E773. Yellow circle indicated implanted embryo.

All blastocysts were cryopreserved following trophectoderm biopsy. Following biopsy genotyping, one blastocyst obtained from mRNA/hyb-gRNA injection (embryo 190; E190) and one from Nuc/sgRNA injection (E773) were transferred into one recipient female rhesus macaque. DNA sequencing of the trophectoderm biopsy revealed that 92.1% of the sequencing reads for E190 possessed a single G insertion that would lead to a frameshift mutation. Sequencing of DNA obtained from the trophectoderm biopsy of E773 revealed that 98.5% of the sequencing reads possessed a 17 bp insertion/50 bp deletion, which would lead to an in-frame 18 bp deletion (Fig. 2a). Additional single embryo transfers (embryos E180, E1170, E1171, and E1289) were performed (n = 4). TE cell analysis showed that E180 carried 99.6% of two different mutations resulting in premature stop codons and 0.2% of wild-type sequence. Embryos, E1170, E1171, and E1289, did not carry any wild-type sequence and all contained frameshift mutations (Supplementary Fig. 2). Out of 5 embryo transfers, a successful singleton pregnancy resulted from the transfer of E190 and E773 into one recipient (20% live birth rate per transfer; Fig. 2b and Supplementary Table 1).

**Figure 3:**
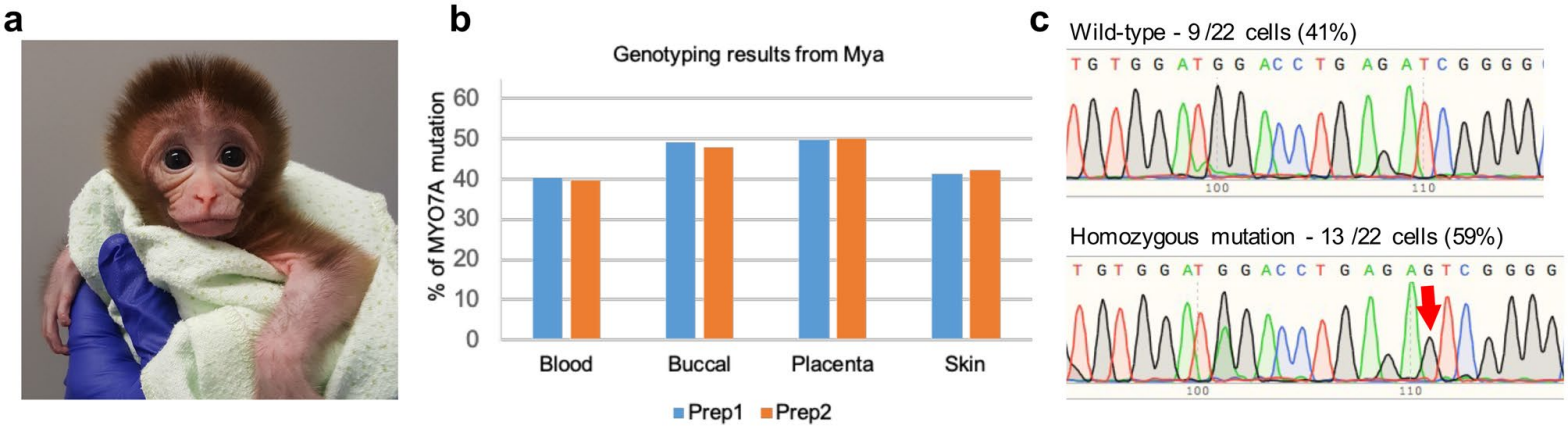
*MYO7A* mutant rhesus macaque infant and single cell genotyping results. (**a**) A healthy female rhesus macaque infant was delivered on day 150 of gestation following the transfer of two *MYO7A* edited embryos. (**b**) Genomic DNA was isolated from duplicate blood, buccal, placenta, and skin samples. Exon 3 of *MYO7A* was PCR amplified and the PCR amplicons were sequenced by NGS. In all tissues, 40–50% of the sequencing reads possessed a single G insertion, confirming the pregnancy was the result of implantation of E190. (**c**) Genotyping results from individual FACs-sorted leukocytes demonstrated that Mya is homozygous mosaic for a G insertion. The top panel shows wild-type *MYO7A* sequence was present in 9 out of 22 cells, whereas the bottom panel indicates a homozygous G insertion (red arrow) was detected in 13 out of 22 individual cells (59%).

A female infant (“Mya”) was delivered by C-section at 150 days gestation (typical gestational length is 158 to 173 days). Mya’s birth weight was 390 g, which was lower than the full-term average of 488 g^22^. Within minutes of delivery, Mya was breathing on her own and appeared healthy (Fig. 3a). Four different tissue samples, including placenta, blood, skin, and buccal specimens, were collected at or within 3 weeks after birth to identify the origin of the transferred embryo based on the *MYO7A* mutation and to assess the level of editing in the offspring relative to what was observed in the TE biopsy (Fig. 3b). The genotyping results from collected samples showed a ‘G’ insertion, confirming that the pregnancy was the result of implantation of E190. In the tissues sampled, the percentage of mutant sequence relative to wild-type *MYO7A* sequence was 40–50%, depending on the tissue analyzed (two biological replicates), compared to the 92.1% mutant *MYO7A* sequence observed in the TE biopsy. To ensure that a larger deletion of the entire exon 3 did not occur, which would be missed by the PCR primers that recognize the sequence directly adjacent to the gRNA target region, primers were used to amplify the entire *MYO7A* exon 3 and adjacent introns in genomic DNA obtained from Mya. No larger deletions encompassing all of exon 3 were observed (Supplementary Fig. 3). Moreover, only a single edit (1 bp insertion) was observed from the sequencing results of the larger exon 3 amplicon, further suggesting additional larger deletions (i.e., deletion of exon 3) did not occur.

The observed level of editing being close to 50% could be due to either editing of one *MYO7A* allele in the one-cell stage (e.g., heterozygous) or homozygous editing in one cell of a 2-cell embryo (e.g., homozygous mosaic). To distinguish between these possibilities, single leukocytes were isolated from a blood sample by FACS and analyzed for the presence or absence of the introduced G insertion following WGA, PCR, and Sanger sequencing. A total of 13 individual cells (59%) were homozygous for the G insertion, whereas 9 cells (41%) had wild-type sequence, confirming that Mya is mosaic for a homozygous frameshift mutation in the *MYO7A* gene (Fig. 3c).

### Assessment of off-target editing

To determine if off-target editing occurred, regions of the rhesus macaque genome with the highest level of homology to the *MYO7A* gRNA recognition sites were chosen for analysis, where each potential editing site had 2–4 bp mismatches relative to the target sequence of each of the gRNAs (Supplementary Table 2). Based on these criteria, 9 sites in the rhesus macaque genome were chosen for further analysis. DNA was extracted from blood, skin, placenta, and buccal cells collected from Mya. Putative off-target sites were PCR amplified and sequenced. Sequencing results revealed that 99.3% of sequence reads corresponded to the wild-type sequence, while < 0.7% possessed a sequence that differed from the reference *MYO7A* sequence. However, these differences were located outside of the sgRNA target sequence, indicating that they represent naturally occurring variants or PCR/sequencing errors. Comparing the region of the amplicon sequences that correspond to the region of homology with the gRNAs, no detectable off-target mutations were observed at the nine sites analyzed (Supplementary Fig. 4).

### Comparison of target gene editing within the rhesus macaque embryo relative to its TE biopsy

Based on the discrepancy in the level of editing observed in the TE biopsy versus tissues obtained from Mya (92.1% versus 59%, respectively), additional sequencing was conducted to assess the accuracy of TE biopsy sequence data in predicting the genotype of the corresponding embryo. Genotyping results between biopsied samples and blastocysts were found to vary (Table 2). The TE biopsy of E1172 possessed 54.42% wild-type sequence reads and 41.65% sequencing reads that included a 70 bp deletion. Sequence analysis of the corresponding blastocyst DNA revealed a lower percentage of wild-type *MYO7A* sequence reads (21.1%). Moreover, the remaining 79.9% sequence corresponded to 8 different indels, but not the 70 bp deletion observed in the biopsied TE sample. The TE biopsy from E1851 sequencing possessed 90.65% of a 1 bp indel and 2.18% of the wild-type sequence. However, the corresponding blastocyst sequencing possessed 42.86% wild-type sequence and 57.15% contained 3 different mutations from TE biopsy result. The sequencing results of TE cells from E1850 also possessed a mixture of wild-type and *MYO7A* edited sequences, of which 12.04% were wild-type sequence and 84.17% included a 1 bp insertion. The blastocyst, however, carried no wild-type sequence and two mutations that differed from what was observed in the TE biopsy. In other instances, greater predictability of the overall level of indels in the biopsy related to the rest of the embryo. For example, E1166 TE biopsy possessed 94.5% wild-type sequence and a mutation was not detected, whereas the remainder of the embryo had 84.84% wild-type sequence along with 3.74% edited sequence that included a 2 bp insertion. The TE biopsy for E1285 had 52.4% wild-type sequence and 45.78% sequence reads with a 1 bp insertion, whereas the remainder of the embryo carried 61.67% wild-type and 30.59% with 4 different mutations. Only 10.71% of the same 1 bp insertion was detected in the embryo. In E1850, the biopsied TE cells possessed 12.04% wild-type and 84.17% of an edited *MYO7A* sequence, whereas the blastocyst had 93.1% sequence showing two different mutations.

**Table 2:**
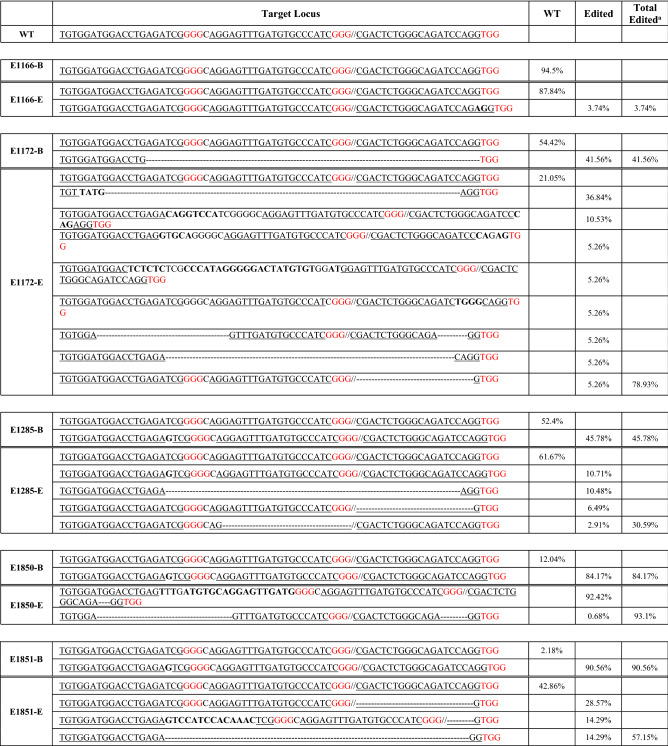
Genotype comparison between the TE biopsy and the corresponding embryo. “B” denotes biopsied sample, and “E” indicates the corresponding remaining embryo. WT refers to the *MYO7A* wild-type sequence. Bold letters indicate insertions and dashes represent deletions. The underline indicates gRNA protospacer sequence and red letters represent the PAM sequence.

^a^WT and Edited sequence percentages do not necessarily add up to 100% because some sequencing results included low quality reads or other sequencing artifacts not aligning to the correct rhesus macaque *MYO7A* reference sequence.

### Evaluation of auditory function

**Figure 4:**
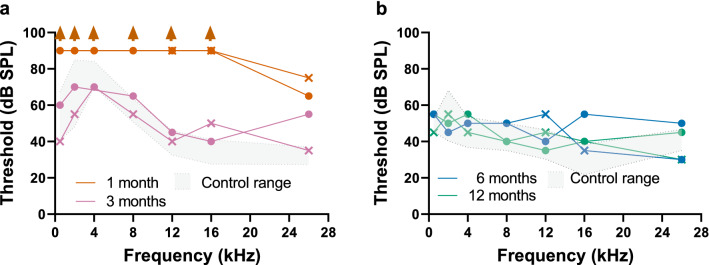
ABR thresholds at 1, 3, 6 and 12 months. (**a**) ABR thresholds in left (circle) and right (X) ears at one (gold line) and three (magenta line) months. Arrows indicate that the threshold, if present, is above 85 dB SPL, the highest stimulus intensity tested. The control range was defined using an in-ear acoustic probe (see “Methods”). (**b**) ABR thresholds in left and right ears at 6 (blue trace) and 12 (green trace) months. The control range was defined using an open field paradigm (see “Methods”).

**Figure 5:**
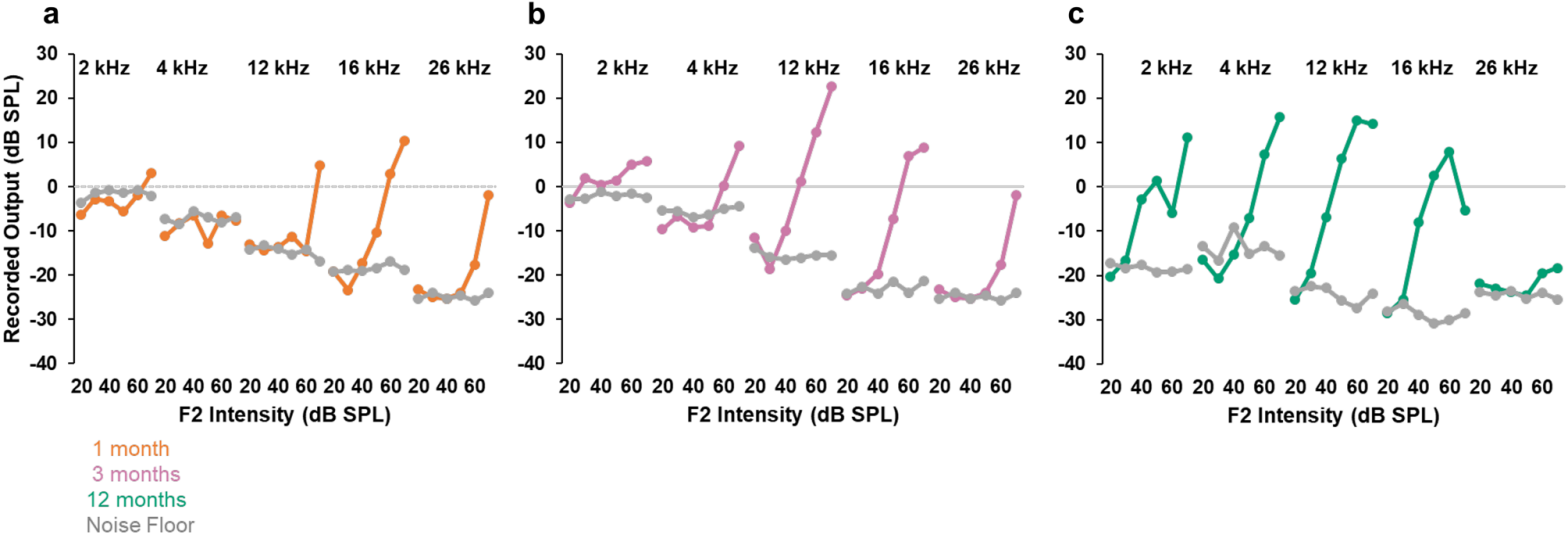
Distortion product otoacoustic emissions at 1, 3, and 12 months. DPOAE responses (2f1–f2; colored traces) at 2, 4, 12, 16 and 26 kHz at one month (gold trace), 3 months (magenta trace), and 12 months (green trace). DPOAE responses were recorded at 6 intensities from 20 to 70 dB SPL incremented in 10 dB steps. The noise floor is in grey for each test age. An authentic DPOAE response profile is characterized by an increase in the magnitude of the distortion product with increasing stimulus intensity.

To assess hearing function in Mya, we measured auditory brainstem responses (ABR) and distortion product otoacoustic emissions (DPOAE) measures at one month of age. No ABR thresholds were detected from 0.5 to 16 kHz (Fig. 4a), and no DPOAE responses were observed from 2 to 12 kHz (Fig. 5a), which is consistent with the phenotype predicted for a null *MYO7A* mutation. However, 26 kHz ABR thresholds were present bilaterally at one month, though dramatically elevated compared to controls (Fig. 4a). Similarly, DPOAE responses that increased in amplitude with increasing stimulus intensity were present at 16 and 26 kHz (Fig. 5a). These high frequency ABR and DPOAE responses suggested that Mya retained some restricted auditory function. Subsequent longitudinal analyses indicated that Mya had sensitive ABR thresholds from 0.5 to 26 kHz at 3, 6, and 12 months (Fig. 4) and robust DPOAE responses from 2 to 26 kHz at 3 and 12 months (Fig. 5b,c). The data indicate that Mya has sufficient functional inner and outer hair cells that support sensitive hearing compared to age-matched controls.

### Evaluation of retinal structure and function

**Figure 6:**
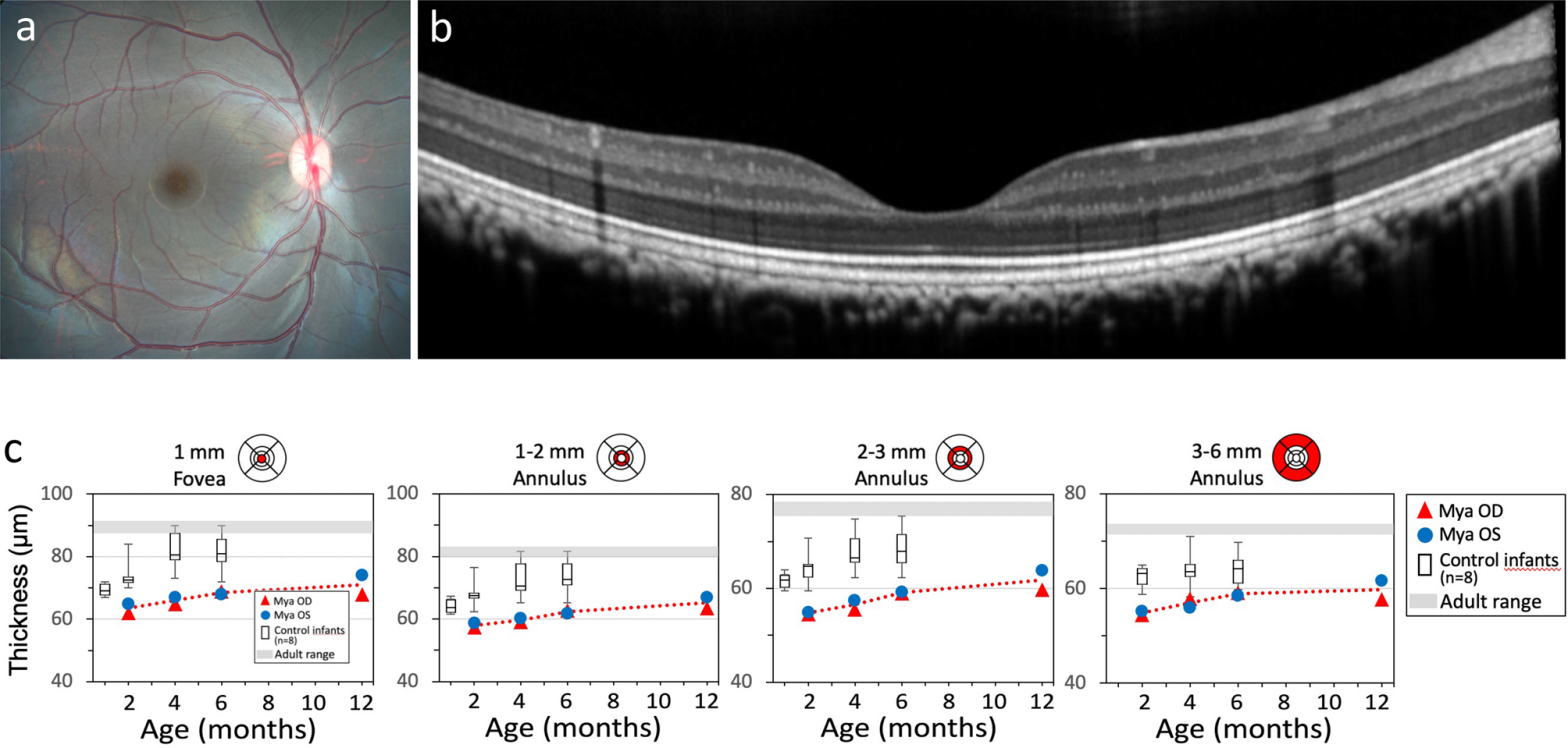
Retinal imaging and retinal layer thickness at 2, 4, and 6 months. (**a**) Color fundus photograph and (**b**) macular sdOCT scan of Mya at 6 months of age, showing normal retinal morphology. (**c**) The thickness of inner + outer segment layers (in µm) in right and left eye of Mya at 2, 4, 6 and 12 months of age, in comparison to data for 8 normal rhesus monkey infants (box plots show mean, interquartile range and range of values for the normal infants). Values were slightly below the normal range in the central 1 mm (fovea) and in each concentric annulus (1–2, 2–3, and 3–6 mm diameter annuli, centered on the fovea). Thickness of the total retina, inner retinal layers and outer nuclear layer were within the normal range except within the fovea, where total retinal thickness was just below the normal range (not shown).

Multimodal retinal imaging, conducted at 2, 4, 6, 9, and 12 months of age, included color fundus photography, spectral domain ocular coherence tomography (sdOCT), fundus autofluorescence (FAF) imaging, ultra-widefield imaging and fluorescein and indocyanine angiography. No pathological features were observed in any imaging mode at any age. A color fundus photograph and a macular sdOCT scan for Mya at 6 months are shown in Fig. 6a,b. The thicknesses of 11 retinal layers were determined by the segmentation of sdOCT images. The only abnormality detected was in the thickness of the inner and outer segment layer, which was slightly below the normal range throughout the central retina (Fig. 6c). Retinal function was assessed by full-field and multifocal electroretinograms (ERGs) at 2.5, 6, 9 and 12 months. Response amplitudes and timing were similar to those of normal age-matched rhesus macaque infants in both scotopic (dark-adapted) or photopic (light-adapted) conditions, indicating normal rod and cone photoreceptor function at each age (Supplementary Fig. 5).

## Discussion

Direct injection of Cas9 and gRNAs into rhesus macaque zygotes allows for the creation of genetically modified NHPs in a rapid and relatively efficient manner^23^. Rhesus macaques have a relatively long gestational period (~ 165 days) and take 3–4 years to reach sexual maturity. By avoiding the need for chimera generation, direct injection into zygotes would be a more efficient route for generating specific disease models. We showed that high efficiency editing can be achieved through injection of multiple sgRNAs and Cas9 protein into the rhesus macaque zygote. By directly injecting CRISPR/Cas9 into rhesus macaque zygotes, modification of the *MYO7A* gene was achieved and resulted in a live-born female infant (Mya). These studies demonstrate that it is possible to target *MYO7A* for the development of a NHP USH1B model. However, further investigation should be conducted to maximize target gene modification efficiency.

Similar targeting efficiency was observed when several *MYO7A* specific gRNAs were tested in a cell-free DNA cleavage assay. However, validation in rhesus macaque CMMT cells demonstrated that three different sgRNAs possessed varying editing efficiency by themselves, but appeared more robust in generating indels in the *MYO7A* gene when used together. These differing results from the PCR amplicon test and the cell line validation might be explained by differing chromosomal accessibility and organization. PCR amplicons of target regions likely offer unobstructed digestion by CRISPR/Cas9^24^. After observing efficient editing in CMMT cells with the combination of three sgRNAs, studies were initiated to determine editing rates within rhesus macaque zygotes using different gRNA and Cas9 formulations, namely the combination of Cas9 mRNA and a hybrid gRNA system (mRNA/hyb-gRNA) compared to Cas9 nuclease protein and a synthetic single gRNA system (Nuc/sgRNA). On-target editing efficiency in rhesus macaque zygotes with Nuc/sgRNA injection was similar to what was reported in various species^25–27^. Alongside higher editing efficiency, we saw a greater proportion of arrested embryos in Nuc/sgRNA injected embryos relative to those receiving mRNA/hyb-gRNA. Similar effects were observed in channel catfish, where an increased level of editing and lower embryonic developmental rates were associated with increasing Cas9 nuclease and gRNAs concentrations^28^. The tradeoff in increased editing efficiency with reduced blastocyst formation rates may be due to several factors. It is possible that more efficient on-target editing is coupled with more efficient off-target editing of embryonic lethal genes that have a negative impact on embryo development. Moreover, recent research in human embryos revealed a propensity of CRISPR/Cas9 to cause chromosomal rearrangements and higher aneuploidy rates, which in turn may compromise embryonic development^29^. It is possible that the embryo that gave rise to Mya also possessed chromosomal abnormalities. However, Mya’s fetal and postnatal development was similar to unedited control rhesus macaques, indicating there are no substantial chromosomal rearrangements present that would be incompatible with implantation, pregnancy, or normal development.

Analysis of cell biopsies from developing embryos is commonly used to detect aneuploidy and disease-causing mutations in human IVF clinics^30^. To avoid wasting resources by transferring and establishing pregnancies with an unedited or incompletely edited embryo, TE biopsies were used to determine the level of gene editing before CRISPR/Cas9 injected embryos were transferred into surrogates. However, the genotyping of different tissues and cells obtained from Mya indicates that a TE biopsy may not fully reflect the edit or range of edits present in the rest of the embryo. The genotyping result from the biopsied TE cells of Mya (E197) showed that 92% of the reads contained a G insertion, which would lead to a frameshift and a premature stop codon, and 8% of the sequencing reads corresponded to wild-type *MYO7A* sequence. Genotyping results from tissue biopsies collected from Mya, however, showed that there were different levels of mutations within different tissues (i.e., 40% of sequence reads with the G insertion in blood cells versus approximately 50% in cells obtained from a buccal swab). These findings suggested that Mya was mosaic for wild-type and homozygous mutant editing rather than heterozygous because the latter would be expected to give a 50% edited and 50% wild-type sequence in all tissues analyzed. Single peripheral blood leukocytes possessed only the G insertion or wild-type *MYO7A* sequence, thereby confirming that Mya is mosaic for the homozygous mutation. To determine if a large deletion exists that encompasses all of *MYO7A* exon 3, primers (888 bp) were used that amplify the entire exon 3 and portions of the flanking introns. PCR product size and Sanger sequencing indicated there was no large deletion. The possibility of larger chromosomal insertions, deletions, or rearrangements cannot be ruled out. However, due to the normal fetal and neonatal development and behavior of Mya, the likelihood of significant chromosomal abnormalities is low. Based on these results, it is likely that the editing event occurred at the 2-cell stage leading to a mixture of wild-type and homozygous mutant (1 bp insertion) cells in Mya.

Our observations are similar to a previously published report wherein sheep blastocysts that arose from CRISPR/Cas9 injected zygotes were biopsied and the TE biopsy was compared to tissue samples collected from the offspring from successful embryo transfers^31^. Similar to our results, the genotypes of the biopsy and the remaining embryo were less consistent when biopsies contained some intermediate level of editing. However, several embryos (E1166, E1285, and E1850) exhibited a similar ratio of wild-type and mutation alleles in the comparison of the biopsy and remaining embryo. Based on these results, PGT may not be suitable for precise genotyping but rather serves as a general indicator of whether any wild-type allele is present in the embryo. It is understandable that the results obtained from a TE biopsy may not fully represent the editing that occurs in the offspring, considering that a typical TE biopsy contains 10–15 cells, which is less than 5% of the population of a whole embryo^32^. Another viewpoint is that if genetic modification happens in the later stage of the embryo (2, 4, 8 cell stages), the accuracy of TE biopsy genotyping is decreased. On the other hand, if editing occurs at the zygote stage, then TE biopsy genotyping provides a more accurate measure of editing. A limitation in our comparison test was that only 5 TE biopsy-embryo pairs were used. Thus, the analyses of additional TE biopsies and embryos will be needed to define the accuracy of using TE biopsies for predicting the level of editing in the corresponding embryo.

Off-target mutations are a major concern with all programmable endonucleases^33,34^. CRISPR/Cas9 targeting of a specific gene relies on a 20 nt spacer sequence at the 5′ end of the guide RNA that is complementary to the gene of interest, and if the genome is large and variable enough, homologous off-target regions of the genome could be affected^35^. Off-target editing is more likely to happen at sites with 1–5 bp mismatches relative to the gRNA target sequence and distal to the PAM sequence^36,37^. However, this is not absolute because a recent study demonstrated that off-targeting events occurred when mismatches were located at the PAM proximal end (i.e., near or adjacent to the seed sequence)^38^. Genomic DNA from Mya was used to assess a total of nine regions of the rhesus macaque genome that possessed high homology to the three gRNAs that were used in the zygote injections (i.e., the 3 regions of greatest homology for each gRNA). Despite each potential off-target site only possessing 2–3 bp mismatches, no off-targeting events were detected at these sites. It should be noted that whole genome sequencing or other large scale CRISPR off-target analyses (CIRCLE-Seq, GUIDE-seq) were not conducted, therefore, potential off-target mutations cannot be completely ruled out.

If a gene-edited model of USH1B faithfully reproduces the human disease, we would expect profound hearing loss at birth as the first indication of an Usher disease phenotype. Therefore, quantitative assessment of auditory function, including ABR and DPOAEs, are essential to validate hearing loss in a rhesus model. Inner hair cells of the organ of Corti forward almost all of the acoustic information collected by the cochlea to the brain^39^. ABRs are a sensitive measure of auditory function that reflects inner hair cell activity^40^. Outer hair cells amplify low intensity sounds and the robustness of DPOAEs reflect outer hair cell dynamics^40–42^. A null mutation in *MYO7A* would be expected to cause gross perturbation of inner and outer hair cell structure, leading to hair cell death and the absence of ABR and DPOAE responses. At one month, we were unable to detect ABR responses from 0.5 to 16 kHz, or DPOAE responses from 2 to 12 kHz in Mya (Fig. 5a). Surprisingly, we detected bilateral, though grossly elevated, ABR thresholds at 26 kHz and sensitive DPOAEs at 16 and 26 kHz in Mya at one month. One explanation for the mixed hearing loss is mosaic *MYO7A* expression in inner and outer hair cells that differentially affects the apex through the middle turn of the cochlea, which subserves low and intermediate frequency responses^43^. While plausible, subsequent detection of sensitive ABR thresholds from 0.5 to 26 kHz at 3, 6, and 12 months, and robust DPOAE responses from 2 to 26 kHz at 3 and 12 months, demonstrated that Mya had sensitive hearing compared to age-matched controls. If Mya is mosaic for *MYO7A* expression in sensory hair cells, the extent of hair cell compromise is insufficient to grossly affect auditory function. Although unedited, full-term controls exhibited normal auditory responses at one month of age, the inability to detect ABR and DPOAE responses in Mya may be anatomic or developmental. Since Mya was born 15 days early, the lack of response may be more related to the narrowness of the neonatal rhesus macaque ear canal that complicates acoustic probe placement. In this constraining anatomical context, it cannot be ruled out that inefficient transmission of pure tone stimuli may have confounded both the ABR and DPOAE measures at one month of age.

Most forms of retinitis pigmentosa, including Usher syndrome, are characterized by initial loss of peripheral rod photoreceptors, leading to night blindness and progressive constriction of the visual field. The time course of retinal degeneration in Usher syndrome is variable, but human children with USH1B show a reduction in the scotopic electroretinogram (ERGs), indicating a reduced function of rod photoreceptors, as early as 6 months^44–46^, which is equivalent to 6 weeks in macaque infants. In contrast, in Mya, both rod and cone function appeared normal through 12 months of age. The only suggestion of abnormal retinal development was a slight reduction in the thickness of the inner and outer segments of the photoreceptors in the central retina, as seen in macular sdOCT scans. Current, generally available OCT imaging technology allows for measures of retinal thickness only in the central retina, not in the periphery where degeneration begins. Therefore, without retinal histopathological analysis, we cannot exclude the possibility of subtle degenerative changes in the peripheral retina in the case of mosaic editing like Mya. It remains to be seen if Mya will exhibit signs of retinitis pigmentosa later in life. However, the lack of a USH1B phenotype in Mya has clinical relevance as it indicates that maintaining or achieving a population of ~ 50% of cells with functional MyoVIIA would suffice in treating individuals with USH1B. Also, the level of *MYO7A* mutation in the sensory organs should be determined in future studies, because normal hearing and vision could be the result of greater numbers of cells that lack the *MYO7A* mutation in the eye and inner ear.

## Materials and methods

### Ethical approval

All protocols involving animals were approved by the ONPRC IACUC and conducted in accordance with the NIH Guidelines for the Care and Use of Laboratory Animals and ARRIVE guidelines. General care and animal housing was previously described^47,48^.

### gRNA design

Web-based algorithms (CRISPOR, Synthego, and IDT) were used to design 4 gRNAs targeting exon 3 of the *MYO7A* gene with optimal on-target efficiency and minimal off-target effects^49^. Hyb-gRNAs were purchased from Integrated DNA Technologies, and sgRNAs were synthesized and obtained from Synthego.

### Target efficiency validation

To confirm the targeting efficiency of the 4 gRNAs, *MYO7A* target site amplicon was digested with Cas9 nuclease (New England Biolabs) and hyb-gRNAs. A total of 100 ng of wild-type *MYO7A* PCR amplicon was digested by 8 ng of Cas9 nuclease and four different concentrations (50, 10, 2, and 0.4 ng/μL) of hyb-gRNA. Reactions were incubated for 30 min at 37 °C, 4 μg of RNAase was added to eliminate the hyb-gRNA, and samples were run on a 1.5% agarose gel.

The CMMT cell line was used to test hyb-gRNA efficiencies alone or in combination. At 75–80% confluency, 600 ng of Cas9 plasmid (Addgene, #64324) was transfected using Lipofectamine 2000 (Invitrogen), followed 24 h later with transfection of hyb-gRNAs using RNAiMax (Invitrogen). A total of 6 pmol of hyb-gRNA was used for individual hyb-gRNA transfections while 3 pmol per hyb-gRNA was used for multi hyb-gRNA transfections. Genomic DNA was obtained (QuickExtract, Epicentre) 2–3 days after transfection. The *MYO7A* target region was amplified and subjected to a T7 endonuclease 1 (T7E1, New England Biolabs) assay according to the manufacturer’s protocol using 200 ng of target amplicon or 150 ng target amplicon combined with 50 ng of wild-type amplicon were used because the inclusion of WT amplicon will ensure the formation of dsDNA containing mismatches that result from Cas9 editing and easily discernable T7E1 cleavage products by gel electrophoresis.

### Zygote injection of CRISPR/Cas9

Oocyte collection and in vitro fertilization (IVF) were conducted as previously reported^50^. Zygotes were stripped free of sperm and transferred to warmed TALP-HEPES under oil. Cas9 nuclease and sgRNAs were prepared as described^26^. Cas9 mRNA injection buffer contained 10 mM Tris–HCl, 10 mM NaCl, and 0.1 mM EDTA, whereas Cas9 protein injection buffer omitted the NaCl. Injection materials included 45 ng/μL of hyb-gRNAs (15 ng/μL of each) with 100 ng/μL of Cas9 mRNA (Trilink) or 45 ng/μL sgRNAs (15 ng/μL of each) with 224 ng/μL of Cas9 nuclease (New England Biolabs). Injections were into the cytoplasm and pronucleus when visible. Injected zygotes were then cultured (BO-IVC, IVF Bioscience) under oil in a 6%, 5%, 89% mixture of CO_2_, O_2_, and N_2_ at 37 °C in humidified air, respectively.

### TE biopsy and embryo genotyping

An objective-mounted laser (Hamilton Thorne) was used to biopsy TE cells of blastocysts placed in warmed Biopsy Medium (IVF Bioscience) under oil. The blastocyst was frozen using the global^®^ Blastocyst Fast Freeze^®^ Kit (LifeGlobal Group). Arrested embryos with the zona pellucida removed by acid Tyrode’s solution, TE biopsy samples, and in some instances, biopsied blastocysts were subjected to WGA (Repli-g Single Cell, Qiagen). The resultant DNA was used to PCR amplify the *MYO7A* target region and amplicons were subjected to next-generation sequencing (NGS) using NEXTFLEX^®^ Rapid DNA-Seq Kit (PerkinElmer). The DNA library was sequenced on an Illumina MiSeq platform and sequence reads were assessed for indel mutations (Geneious software).

### Off-target editing analysis

Putative off-target sites were identified using CAS-OFFinder^51^. Off-target regions were selected based on exon regions over introns and intragenic regions with high homology at the seed sequence which is more permissive for off-target editing. Off-target sites were selected for analysis based on the following parameters: they include exons, possess a high degree of homology to the seed sequence, and have less than 4 total mismatches between the gRNA and target sequences. Using these criteria, three potential off-target sites for each gRNA target (Supplementary Table 2) were amplified by PCR. Resultant amplicons were subjected to adapter ligation and sequenced on an Illumina MiSeq platform. CRISPresso2 software was used for sequence analysis^52^.

### Embryo transfer

Embryos selected for transfer were thawed (Global^®^ Blastocyst Thawing Kit, Life Global) and maintained for 2 h in 100 µL drops of BO-IVC under oil to monitor post-thaw viability. Embryos with blastocoel reformation or expansion were transferred into recipients as previously described^53^. A transfer catheter was inserted into the oviduct and the embryo was deposited in 10 µL of TALP-HEPES. After embryo transfer, pregnancy was confirmed by ultrasound at gestational day 28.

### *MYO7A* genotyping

Genomic DNA was extracted from blood, buccal swabs, skin, and placental tissue and used to PCR amplify the targeted region of *MYO7A* (primers detailed in Supplementary Table 3). PCR amplicons were purified and prepared for NGS sequencing (NEXTFLEX^®^ Rapid DNA-Seq Kit) using an Illumina MiSeq platform with roughly 5–10 thousand reads per sample. Geneious software and the CRISPResso pipeline were used for sequence analysis. Single cell genotyping was conducted on leukocytes isolated from whole blood. Using a Becton Dickinson Aria-II FACS, cells were sorted by morphology and then underwent WGA, with the resultant DNA being used for PCR amplification of *MYO7A* exon 3. Purified amplicons were then Sanger sequenced. Exon3 primers were used to detect large deletion on target locus and PCR amplicon was analyzed by Sanger sequencing.

### Assessment of auditory function

The animal was anesthetized and placed within an IAC Acoustics MAC-3 RF-shielded Mini-Acoustical Chamber. The patency of the distal ear canal was confirmed by otoscopic evaluation. Middle ear function was tested at 1 kHz or 226 Hz by the ZODIAC Diagnostic Tympanometer (Model 8-04-16045). For 1-month and 3-month testing, stimuli were delivered in the ear canal via a custom acoustic assembly comprised of two dynamic drivers (Etymotic Research ER4B-SG) and a miniature electret condenser microphone (Knowles FG-23329-P07). For testing at 6- and 12-months, stimuli were delivered with an open field speaker (Fostex FF85WK) calibrated and monitored with a ¼-inch condenser microphone (Larson Davis Model 377C10).

Acoustic stimuli were conducted as previously reported^54^. Five-ms tone pip stimuli with 0.5-ms rise/fall times (cosine squared) in alternating polarity were presented in an interleaved stimulus train from 20 to 85 dB SPL in incremental steps of 5 dB at each frequency tested. The order of frequencies was 26, 12, 4, 0.5, 16, 8 and 2 kHz presented at a stimulus rate of 60/s. The response from the electrodes was amplified 10,000 ×, filtered at 300 Hz–3 kHz band pass, and averaged for 300 samples at each frequency-level combination. Responses were recorded from subcutaneous electrodes located at the vertex, the ipsilateral mastoid, and the shoulder.

DPOAEs were recorded using the ear canal sound delivery apparatus controlled by Eaton Peabody Laboratory Cochlear Function Test Suite software (ver. 2.6.3.831). Primary f2 tones were presented at 2, 4, 12, 16 and 26 kHz with a primary frequency ratio f2/f1 of 1.2 and the f2 primary level 10 dB less than the f1 level. Primaries were incremented in 10 dB steps, with f2 from 20 to 70 dB SPL. Ear-canal sound pressure was amplified, digitally sampled and averaged, Fast Fourier Transforms were computed and averaged, and the DPOAE and noise floor at 2f1–f2 were extracted.

### Assessment of retinal structure and function

Retinal structure was evaluated under isoflurane anesthesia with retinal imaging, including color fundus photography and fluorescein angiography (Zeiss FF450), sdOCT (Heidelberg Spectralis) and quantitative fundus autofluorescence^55^ (Spectralis Blue Peak mode), and ultra-widefield imaging (Optos California)^56–59^. Retinal function was assessed with full-field electroretinography (Espion Diagnosys) under anesthesia with ketamine and xylazine. For each of these measures, results for Mya were compared to those for age-matched normal rhesus infants.

OCT imaging parameters included a standard scan (30° × 25°; ART:20; 61 lines with ~ 120 µm spacing), plus two high density scans (#1:15° × 10°; ART:20; 49 lines with ~ 60 μm spacing; and #2: 15° × 5°; ART:20; 97 lines with ~ 30 μm spacing) for characterization of the macula/fovea and any salient features of interest. The directional OCT method was performed as described to obtain an accurate measurement of the foveal outer nuclear layer^60^. Spectralis segmentation software (EyeExplorer 1.10.4) was used to obtain layer thicknesses, with manual inspection and corrections of each slice. Corneal curvature and axial length were measured with an IOL Master at each retinal imaging session to allow conversion of retinal parameters from degrees of arc to mm. Ultra-wide field imaging in the peripheral retina, including pseudocolor, FAF, fluorescein angiography of the retinal vasculature, and indocyanine angiography of the choroidal vasculature, was performed to identify peripheral changes associated with retinitis pigmentosa^59^. Full-field ERGs were recorded with a Diagnosys Espion system using custom bipolar Burian–Allen contact lens electrodes and included photopic, flicker and scotopic intensity-response series, and a dark adaptation series.

### Statistics analysis

To compare the results of embryo development and target gene editing between the two conditions (mRNA and Protein), we conducted a two-tailed Fisher’s Exact Test. A p value < 0.01 was considered statistically significant.

## Supplementary Information

Supplementary Information.
