# Supplementary material for: CRISPR/Cas9 editing of the MYO7A gene in rhesus macaque embryos to generate a primate model of Usher syndrome type 1B
Source: Sci Rep. 2022 Jun 16;12:10036. doi: 10.1038/s41598-022-13689-x (PMC9203743; doi:10.1038/s41598-022-13689-x)
Supplement: Supplementary file 1 — Supplementary Information. [file 41598_2022_13689_MOESM1_ESM.docx]

**Supplemental Information**

Supplementary Fig 1. Targeting efficiency test of sgRNAs *in vitro*.

Cas9 protein incubated with hyb-gRNA and PCR amplified *MYO7A* exon 3 DNA was digested by 4 different concentrations of Cas9 nuclease (50, 10, 2, and 0.4 ng/μL). The last lane is a non-digested wild-type PCR amplicon.

Supplementary Fig 2: Genotype results for biopsied embryos that were subsequently transferred.

Before embryo transfer, TE cells were biopsied from CRISPR/Cas9 injected blastocysts. Except for embryo E188, the wild-type *MY07A* sequence was not detected. Underlined letters are gRNA sequence, bold ltters indicate random insertion and ‘-‘ indicates deletion. Red letters denote the PAM sequences.

Supplementary Fig 3: Detection of large deletion on *MYO7A* exon 3 region.

(a) To confirm the unexpected large deletion on target loci, exon3 primers were used to amplify the entire exon 3 and flanking intron regions. Genomic DNA was obtained from skin. Primers were located around 400bp away from the target region and large deletion was not detected from Mya. L indicated 100bp ladder, Mya means PCR product from Mya genomic DNA, and N is negative control. (b) Sanger sequencing result indicated Mya carried wild-type sequence and 1bp insertion mutation alleles. The bolded and underlined letter denotes the single bp insertion.

Supplementary Fig 4. Off-target analysis of DNA from Mya.

Using the web-based program, CAS-OFFinder, putative off-target sites were selected. A total of nine sites were amplified and sequenced by NGS. Sequencing results were analyzed using CRISPResso2. Modifications (<0.7%) at expected target sites were detected out of double-strand break region. U.M. indicates wild-type sequence and M indicates mutation sequence.

Supplementary Fig 5. Retinal function at 6 months

(a) Full-field ERG of Mya at 6 months of age, showing photopic (light-adapted) and scotopic (dark-adapted) waveforms with similar amplitude and timing to those of a representative 12-month-old normal infant (bottom row). Amplitudes (b) and peak latencies (c) for a-wave and b-wave components of the photopic (left) and scotopic (right) ERG for Mya at 2, 4, 6, 9 and 12 months of age (red triangles and dotted lines) in comparison to mean ± standard error values for 8 normal infants (back open squares). Mya showed no consistent abnormalities in any of these measures.

Supplementary Table 1. Summary of oocyte and sperm donors, recipient, and embryo transfer animals.

A total of 5 embryo transfers were conducted resulting in one pregnancy.

| Oocyte donor ID | Sperm donor ID | Recipient ID | Transferred embryo | Pregnancy | Outcome |
| --- | --- | --- | --- | --- | --- |
| 02 | 64 | 62 | E190 | Yes | 13(E190) |
| 91 | 83 |  | E733 | No |  |
| 91 | 83 | 20 | E188 | No |  |
| 99 | 64 | 67 | E1170 | No |  |
| 99 | 64 | 20 | E1171 | No |  |
| 63 | 64 | 03 | E1289 | No |  |

Supplementary Table 2. Potential off-target sites and locations.

Red letters indicate mismatch against gRNA *MYO7A* target sequences. Red letters indicate mismatched sequence between the gRNA protospacer sequence.

| sgRNA | # | Off-Target Sequence (5’ – 3’) | Location |
| --- | --- | --- | --- |
| MYO7A-1 | A | TGTGGAGGGACCTGAGATGG | CM002977.3, 21700205-21700227 |
|  | B | TGTGGATGGACCTGAGAATG | CM002986.2, 10584900-10584922 |
|  | C | TGTAGTTGGACCTGAGAACG | CM002996.3, 12610193-12610215 |
| MYO7A-2 | A | TGGACTTTTATGTGCCCATC | CM002984.2, 33542558-33542580 |
|  | B | AGGAGTTTTCTGTGCCCATG | CM002978.2, 92883746-92883768 |
|  | C | AGGAGTTTGATTTGTCCTTC | CM002982.3, 71871922-71871944 |
| MYO7A-3 | A | TGACTCTGGGCTGATCCAGG | CM002983.2, 78995551-78995573 |
|  | B | CGTCTCAGGGCAGCGCCAGG | CM002996.3, 49919241-49919263 |
|  | C | AGACGCTGGGCAGATCCTGG | CM002980.3, 79918789-79918811 |

Supplementary Table 3. Primer information.

Primers used in this study for amplifying the *MYO7A* target region and putative off-target regions.

| Primer | Primer sequence (5’ - 3’) |
| --- | --- |
| MYO7A-NGS-F | TTCCCCCTTCTGTGGTTGGC |
| MYO7A-NGS-R | GAGGGGACTACTCACATTGCCT |
| MYO7A-exon3-F | GAGTGGTGTCCCTGTGAGGA |
| MYO7A-exon3-R | GTCTGAGGAGCTTCTAGCCTG |
| sgRNA1-offtarget1-F | TGTGTACATGTGGGTTTACTG |
| sgRNA1-offtarget1-R | CAGCAATTTGAAGAAACCTGC |
| sgRNA1-offtarget2-F | GATATTGTGGGGCGTGGTAACTC |
| sgRNA1-offtarget2-R | TTTTGGAGAAATTGGGAGGAAAGC |
| sgRNA1-offtarget3-F | TTCTTGACTTCACCAAAGACTGGG |
| sgRNA1-offtarget3-R | GAACCTGCTGGGCATGATCTTC |
| sgRNA2-offtarget1-F | GACTTTGAAACATAGCTCTTGG |
| sgRNA2-offtarget1-R | GTTTCTGCAAAATCTGAAGCTG |
| sgRNA2-offtarget2-F | TTGTAGGTCTGTCCCAAGTC |
| sgRNA2-offtarget2-R | CACTTTGTTTTGTCACAGTCG |
| sgRNA2-offtarget3-F | ATGTTTTGTGCTTGCCCTTG |
| sgRNA2-offtarget3-R | ACAGCCTATTTATTTGGAGAGG |
| sgRNA3-offtarget1-F | GCTATCATTTTACTGGAGTGTTG |
| sgRNA3-offtarget1-R | CAAGATTCTCCTGTAGTAAGAAC |
| sgRNA3-offtarget2-F | TGCTGATCTTCCTGTGTCTCTG |
| sgRNA3-offtarget2-R | TGTACCAGGCTGAATTTCCC |
| sgRNA3-offtarget3-F | CACCATGTATTGTCATTGGC |
| sgRNA3-offtarget3-R | GCACTATGGGAGTGTGATTAG |

Figure 1b.

Supplementary Fig 1.


Supplementary Fig 3.
